# Bone marrow vasculature advanced *in vitro* models for cancer and cardiovascular research

**DOI:** 10.3389/fcvm.2023.1261849

**Published:** 2023-10-17

**Authors:** Marzia Campanile, Leonardo Bettinelli, Camilla Cerutti, Gaia Spinetti

**Affiliations:** ^1^ Laboratory of Cardiovascular Research, IRCCS MultiMedica, Milan, Italy; ^2^ Department of Experimental Oncology, IRCCS-IEO, European Institute of Oncology, Milan, Italy.

**Keywords:** bone marrow vasculature, *in vitro* models, HSPC mobilization, cancer, diabetes

## Abstract

Cardiometabolic diseases and cancer are among the most common diseases worldwide and are a serious concern to the healthcare system. These conditions, apparently distant, share common molecular and cellular determinants, that can represent targets for preventive and therapeutic approaches. The bone marrow plays an important role in this context as it is the main source of cells involved in cardiovascular regeneration, and one of the main sites of liquid and solid tumor metastasis, both characterized by the cellular trafficking across the bone marrow vasculature. The bone marrow vasculature has been widely studied in animal models, however, it is clear the need for human-specific *in vitro* models, that resemble the bone vasculature lined by endothelial cells to study the molecular mechanisms governing cell trafficking. In this review, we summarized the current knowledge on *in vitro* models of bone marrow vasculature developed for cardiovascular and cancer research.

## Graphical Abstract

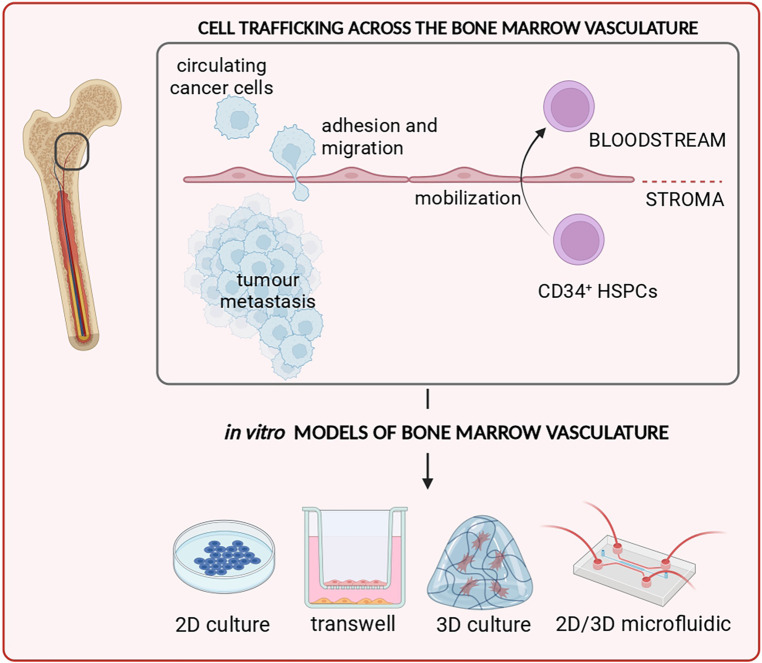

## 1. Introduction

Cardiovascular diseases (CVDs) and cancer are among the leading causes of death in Western countries (1, 2) and together account for over 60% of all deaths in Europe (3). CVDs may arise as a severe complication of diabetes (4) that, being largely diffuse in the global population (5), constitute a serious concern in the cardiovascular field. As for solid cancers such as breast cancer, thanks to treatment improvements and early diagnosis, primary tumors are largely treatable, however with longer life expectancy, cancer recurrence and secondary tumor (metastasis) incidence exponentially increase and are the cause of 90% of cancer-related death (6). In this context, the bone marrow (BM) represents a critical organ in both cardiovascular and cancer research.

The BM resides in the bone cavities and is deputed to new blood cell generation and release in the bloodstream; therefore it is characterized by high cellular trafficking both in health and disease. Moreover, the BM releases stem cells directing tissue regeneration in response to cardiovascular damage, as following myocardial infarction (7). The term “mobilopathy” was coined to indicate the limited CD34^+^ hematopoietic stem progenitor cell (HSPC) inside-out migration (8) observed in diabetic patients and associated with poor CVDs outcome (9–13). To facilitate cell migration to the bloodstream the BM vasculature presents a fenestrated endothelium at the capillary level, but it may also provide easier access to cancer cells (14–16). The bone is also the primary site where blood cancer begins, such as leukemia, and a site for solid tumors metastasis formation, such as breast cancer (17). Interestingly, numerous studies identified a cross-talk between CVDs and abnormal hematopoiesis, and the central role of BM endothelial cells (ECs) in this process was assessed. CVDs induce phenotypic alteration of ECs, which modify their inflammatory citokynes profile inducing changes in the HSPCs proliferation rate (18). On the other hand, an enhanced myelopoiesis has been linked to CVDs such as atherosclerosis (19).

The understanding of the mechanisms that control hematopoiesis and cell's trafficking from and to the BM, both in health and diseases is increasing the attention from clinical and basic researchers since they may pave the way to new preventive and therapeutic strategies in the clinic. The structure of human bone marrow vasculature is well known, but the majority of our knowledge of the molecular mechanisms of cellular trafficking derives from animals, which are known to not be able to fully mirroring human physiology (20). Therefore, to move toward more clinically relevant models, new *in vitro* cultures have been developed. The combination of these advanced *in vitro* tools with human-derived cells will represent a step forward for mechanistic studies and for personalized medicine applications to identify prognostic and therapeutic targets.

## 2. Bone marrow structure and function

**Figure 1:**
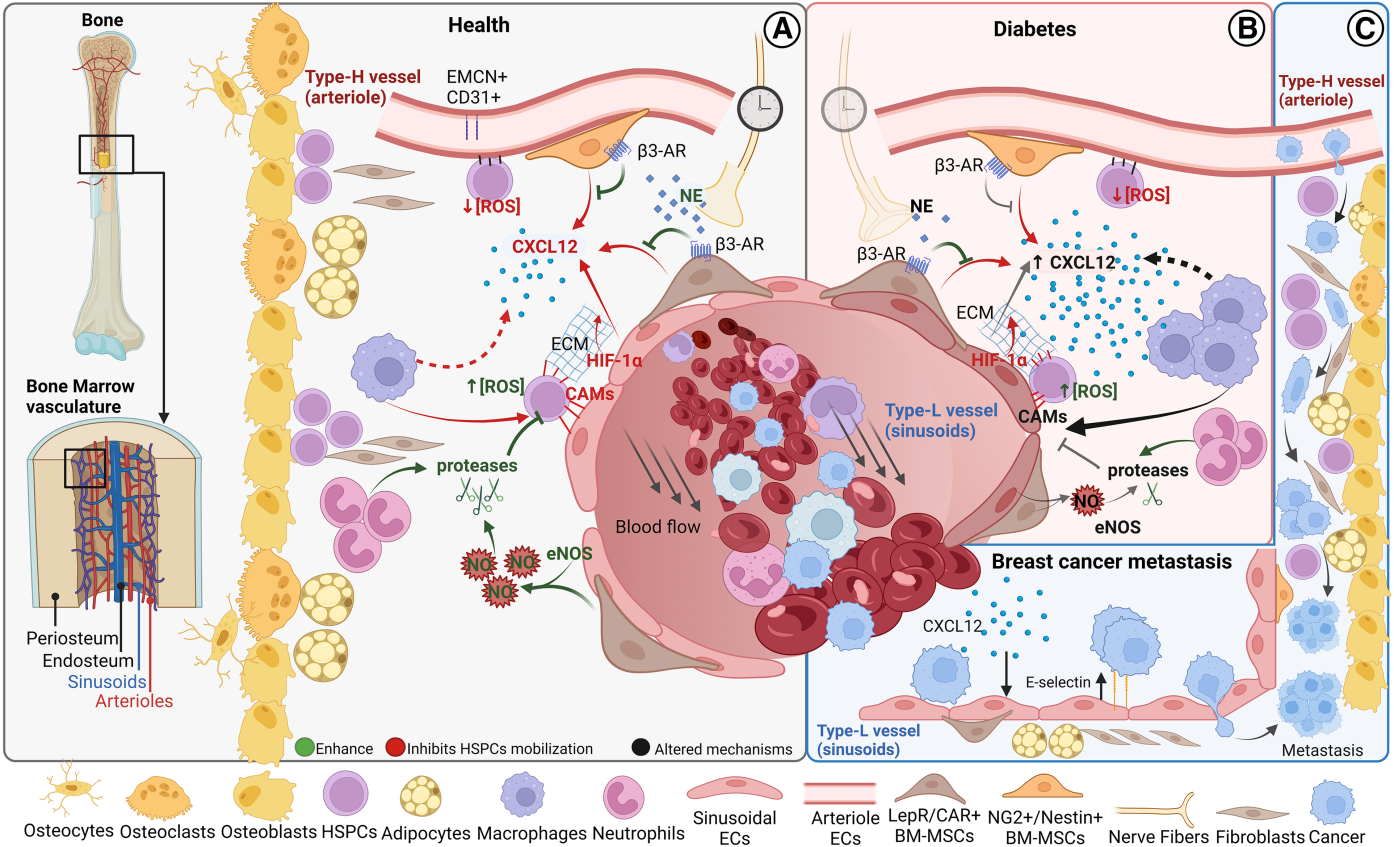
Bone marrow cellular composition and main vascular related trafficking mechanisms in homeostasis and disease. Schematic of BM localization and the cellular components **(A)** BM cell trafficking mechanisms in homeostasis. **(B)** Altered cell trafficking mechanisms in diabetes **(C)** Hypothesized steps of cell trafficking in breast cancer bone metastasis formation. BM, bone marrow; HSPCs, hematopoietic stem progenitor cells; CAMs, cell adhesion molecules; CXCL12, CXC chemokine ligand 12; PSCs, perivascular stromal cells; ECs, endothelial cells; NE, norepinephrine; AR, adrenoreceptor; NO, nitric oxide. Figure realized with www.biorender.com.

The BM is a vascularized tissue composed of a variety of cell types contributing to its homeostasis and function, including HSPCs, bone marrow mesenchymal stem/stromal cells (BM-MSCs), osteoblasts, osteoclasts, adipocytes, lymphatic and vascular endothelial cells (ECs), and immune cells (21) (Figure 1). The BM's main function is to generate mature blood cells during hematopoiesis, a process that leads to the generation of the different cellular components of the immune system that can traffic in and out of the BM through its vasculature. Common myeloid and lymphoid progenitors generate from the partial differentiation of multipotent progenitors, which are transient-amplifying cells, originating from the asymmetric division of short-term hematopoietic stem cells (ST-HSCs) (22).

The myeloid progenitors differentiate in erythrocytes, granulocytes (basophils, neutrophils, and eosinophils), monocytes, and megakaryocytes (platelets-producing cells), while lymphoid progenitors in lymphocytes (T-cells, B-cells, and natural killer cells) (23). Adult BM-MSCs are a heterogeneous population of multipotent cells (24) with the ability to differentiate into cells of mesenchymal origin such as adipocytes, chondrocytes, and osteoblasts when exposed to specific growth signals (25, 26) that are forming the BM. Osteoblasts and osteoclasts are the main cell types involved in bone homeostasis and its repair upon injury. Osteoblasts regulate bone formation, and secrete the extracellular matrix (ECM) proteins such as type I collagen, osteopontin, osteocalcin, and alkaline phosphatase, important components of the BM structure, while osteoclast are large multinucleate cells, originating from the hematopoietic lineage, manly involved in bone resorption (27). BM adipocytes are responsible for filling the medullary canal of long bones (tibia, femur, humerus), and gradually increasing their presence in the marrow during lifespan depending on marrow type (28).

### 2.1. Bone marrow vascular niche structure and function

HSPCs are organized in specialized BM microenvironments, called “BM niches”, that regulate HSPC self-renewal, differentiation, and mobilization (29–32). Two types of BM niches have been identified based on their localization: the endosteal and the perivascular niche which could be further classified as periarterial and perisinusoidal (33) (Figure 1).

Each niche is characterized by specific oxygen levels, cellular components, and soluble factors that determine a differential modulation of HSPC activity (33, 34). The function of each niche has yet to be fully clarified and the topic remains controversial (33, 35, 36).

It is now well established that the majority of HSPCs localize near the vasculature (37–42), lined by BM endothelial cells (BMECs), but it is still debated whether HSPCs are mostly associated with sinusoids or arterioles (37, 41, 43). Blood vessels are distributed throughout the bone tissue except in cartilaginous areas such as the growth plate. The bone marrow vasculature, mostly consisting of dense and highly branched sinusoidal vessels, provides the barrier between the hematopoietic compartment and the peripheral circulation (15, 44). Sinusoids are also called type L vessels and are mainly located in the diaphysis of long bones, whereas arteriolar type H vessels are characteristic of metaphysis (37, 45, 46). Type H vessels, which are fed directly by arterioles and, therefore, exhibit higher partial pressure of oxygen (pO2) and blood velocity than type L vessels, are interconnected at their distal ends, near the growth plate, by structures termed loops or arches (44). Sinusoids are larger and fenestrated, resulting in much more permeable vessels compared to arterioles (15). The higher permeability is associated with a higher reactive oxygen species (ROS) state of HSPCs (34) (Figure 1), which mediates HSPC mobilization (47, 48). The work of Itkin et al. demonstrated that HSPC migration toward the bloodstream occurs exclusively at sinusoids (34). Importantly, sinusoidal and columnar vessels are interconnected and form a single vascular network (44).

Type H ECs express higher levels of endothelial markers CD31 (PECAM1) and endomucin compared to type L ECs (45, 46). Type H vessels couple angiogenesis and osteogenesis and regulate bone development in growing bones. Type H vessels are surrounded by a large number of osteoprogenitors and secrete osteogenic factors, such as platelet-derived growth factors (PDGFs), fibroblast growth factor1 (FGF1), and transforming growth factors (TGFs), to support osteoprogenitor cell survival and proliferation, providing a resource of osteoblasts for bone formation. Type H vessels also support vessel-associated osteoclasts through a RANKL–RANK signaling pathway, promoting cartilage resorption and directional bone formation (49).

Beyond the type of vessel and ECs, different perivascular BM-MSCs discriminate the periarterial from perisinusoidal niche. Around arterioles, BM-MSCs are positive to Neuron Glial 2 (NG2) and Nestin whereas perisinusoidal BM-MSCs are leptin receptor (LepR)-expressing CXC chemokine ligand 12 (CXCL12)-abundant reticular (CAR) cells (41, 42). Pericytes are defined as perivascular cells distributed along the microvessel walls and behave as mesenchymal stem cells (MSCs) *in vitro* displaying adipogenic, osteogenic, and chondrogenic potential (50). Given this definition, both LepR/CAR cells and NG2^+^/Nestin^+^ cells could be considered pericyte subtypes (50, 51), even if in some studies pericytes have been confined to smooth muscle α-actin (α-SMA) positive cells (52). Other mature hematopoietic cells, such as megakaryocytes (MKs), macrophages, and neutrophils are also present near the vasculature and could be considered part of the perivascular niche (Figure 1) (53).

In this review, we will describe the *in vitro* models of bone marrow vasculature developed so far for cardiovascular and cancer research with a particular focus on cell trafficking of angiogenic BM stem cells and cancer cells across the BM vasculature describing the mechanisms involved. Providing details of the *in vitro* approaches used we aim at highlighting the current limits and the comparative benefits.

## 3. Bone marrow cellular trafficking in cardiovascular and cancer research

### 3.1. Bone marrow HSPCs mobilization mechanisms

BM CD34^+^ stem cells can support neoangiogenesis restoring blood flow and facilitating inflammatory response. They are physiologically mobilized in response to infections and tissue damage (54, 55) and their release can be pharmacologically stimulated too (56). Granulocyte colony-stimulating factor (G-CSF) is the most used substance for BM stem cell mobilization and has been widely used to study the HSPC mobilization process in animal models (56).

G-CSF mobilizes HSPCs through two principal mechanisms (i) reducing BM levels of the chemoattractant chemokine CXCL12 and (ii) disrupting the HSPCs cell adhesion molecules (CAMs)- dependent attachment to the BM microenvironment.

In the BM, CXCL12 is principally released by perivascular BM-MSCs, in a circadian fashion through the norepinephrine/β3-adrenoreceptor signalling pathway (57–59), and ECs (35) (Figure 1). The ECs CXCL12 secretions are required for HSPCs maintenance in the BM niche (35), and hypoxia-inducible factor-1α (HIF-1α) may play a role in this mechanism (60, 61) (Figure 1). Also, macrophages have been linked to HSPC retention in the BM through the CXCL12 signalling (62–64) (Figure 1).

HSPCs are anchored to the BM niche through the expression of CAMs (65) (Figure 1). Macrophage depletion induces HSPC mobilization also reducing the expression of CAMs and CAMs ligands (62). Neutrophil-derived matrix metalloproteinase-9 (MMP-9), neutrophil elastase, and cathepsin-G directly cleave some CAMs *in vitro* (65, 66) highlighting the mechanism by which neutrophils induce HSPCs mobilization in response to G-CSF in treated mice (66–68) MMP-9 activity is modulated by levels of nitric oxide, which is produced by endothelial nitric oxide synthase (eNOS) (69). Suppression of eNOS in BM significantly decreased endothelial progenitor cells' release into the bloodstream (70, 71). In BM eNOS is predominantly expressed in panendothelial cell antigen antibody (MECA32) positive cells, suggesting that MECA32^+^ cells’ expression of eNOS influences HSPCs mobilization through activation of MMP-9 (71). It is still debated the role of the osteogenic niche in cellular mobilization (35, 57, 59, 61, 62, 64, 65).

### 3.2. Altered bone marrow HSPCs mobilization in diabetes

Type 2 diabetes mellitus (T2DM) not only results in altered BM structure and stem cell count (72–74) but also, in most investigations reduced CD34^+^ circulating cells were observed in T2DM (11, 72, 75, 76) and type 1 diabetes (77). A meta-regression analysis shows that G-CSF-induced HSPCs mobilization is inhibited in diabetic patients (78). Indeed, diabetes impairs the stem cell mobilization mechanism at different levels. In diabetic mice, G-CSF administration fails to reduce CXCL12 levels (Figure 1). This abnormality in G-CSF regulation of CXCL12 levels is rescued by direct pharmacologic inhibition of CXCL12-CXCR4 signalling, suggesting that diabetic mobilopathy is caused by impairment in CXCL12 levels regulation and not in HSPCs intrinsically (79). Two studies demonstrated that diabetes deregulates stem cell motility through autonomous nervous system impairment (79, 80) (Figure 1). The laboratory of Paolo Madeddu, MD, demonstrated that substance P/neurokinin 1 receptor (NK1R) nociceptive signalling is implied in HSPCs mobilization and that nociceptive neuropathy reflects the decrease in NK1R-HSPCs circulating cells and reduced recruitment of NK1R-HSPCs to the ischemic site in diabetic model (81, 82).

Vessel degeneration observed in diabetes is thought to be largely caused by ECs dysfunction triggered by hyperglycaemic conditions (83). In the context of the BM niche, ECs express less CXCL12 in diabetic animal models (84) (Figure 1). On the other hand, there is an increase in the number of pro-inflammatory CD169^+^ macrophages, that correlated with HSPCs mobilopathy, in the diabetic mice model compared to wild-type (85).

Moreover, diabetes could potentially increase CAMs levels suppressing eNOS activity (86) (Figure 1), indeed a higher number of VCAM1 positive HSPCs have been observed in diabetic mice (87).

*In vitro* models mimicking BM vasculature-CD34^+^ cell interaction could allow a deeper understanding of the aforementioned mechanisms of cellular mobilization depicting the target mechanisms to enhance it in conditions in which it is impaired.

### 3.3. Bone marrow cancer metastasis

The BM vascular niche plays a crucial role in the development and progression of hematologic (88, 89), and solid cancer (90). Despite the medical progress in primary tumor treatments, cancer metastasis is the leading cause of cancer-related death. Metastasis is a complex process that involves several stages, including the invasion of surrounding tissue by cancer cells, entry into the bloodstream or lymphatic system, and eventual attachment to the target organ endothelium, where circulating tumor cells (CTCs) may arrest. CTCs that firmly adhere to ECs can form metastases by squeezing through small gaps between ECs and entering the secondary tissue via cell extravasation. Once in the tissue, disseminated tumor cells (DTCs) may establish a new colony by multiplying and growing, combined with new blood vessel formation to support their growth. Breast cancer is the most common cancer in women worldwide and has a high propensity to form metastasis in the bone. After years of latency, 70%–90% of the patients presented bone lesions (91). Breast CTCs are released from the primary tumor and subtypes of resistant cells reach the BM long before the metastasis formation. Oestrogen receptor (ER), progesterone receptor, and human epidermal growth factor receptor 2 (HER2) triple-positive breast cancer subtypes have a high risk of bone metastasis (92). Recent evidence suggests that the interaction between epithelial to mesenchymal transition (EMT) transcription factors like Zinc Finger E-Box Binding Homeobox 1 (ZEB1) and ERα promote breast cancer bone metastasis (93). The precise mechanisms by which CTCs migrate within the bone marrow are not yet fully understood. It has been hypothesized that breast CTCs migrate across the BM sinusoidal walls, invade and survive in the BM stroma, and then reach the endosteal niche, rich in arterioles type H-vessels, the site for breast cancer bone metastasis (90, 94) (Figure 1). Type H-vessel ECs express chemokines like CXCL12 and adhesion molecule E-selectin that induce DTCs migration, retention, and activation across the type H vessels (95–97) (Figure 1). Breast DTCs remodel the BM niche and type H vessels to support metastasis by releasing factors like G-CSF (98). These events lead to breast bone metastasis, that are mainly osteolytic (bone resorption-lytic), but also osteoblastic (increased bone formation) affecting normal BM function and patient poor outcome. Current therapies are still palliative, and few approved therapies are available to patients. Preclinical studies show that E-selectin inhibitor suppresses tumor cell homing to the bone and outgrowth of micrometastasis (91). Despite these recent findings to unravel breast cancer metastasis formation in the BM, further understanding of all metastatic steps is essential for developing effective therapies to prevent or treat breast cancer bone metastasis. As pointed out earlier for BM stem cell mobilization in diabetes, recent advances in *ex-vivo* microfluidic vascular models in combination with emerging BM vascular models might provide insight into the underlying mechanisms of bone metastasis formation and therapeutic targets for effective clinical treatments.

## 4. Bone marrow vascular *in vitro* models

### 4.1. 2D bone marrow *in vitro* models

Traditional 2D cultures and cocultures have been the main systems used in biology, including vascular research. *In vitro* vascular models of BM included both murine and human-derived vascular and BM niche cells cultured under static or dynamic conditions. Many 2D static models of BM vasculature employed non-BM ECs, both in murine and human culture. For example, a rat 2D model of BM was obtained seeding primary BM-MSCs and primary aortic ECs, on the opposite side of a semipermeable membrane (99).

Murine and human-derived cells were used in combination in hybrid models by coculturing the widely used and characterized primary human vein ECs derived from the umbilical cord (HUVECs), representing the vascular endothelium, with primary mouse BM-derived HSPCs (100), or mouse leukemic initiating cells (LICs) (120). Kobayashi et al. also employed human primary ECs isolated from the skin and aorta as vascular components of the bone marrow model (100).

**Figure 2:**
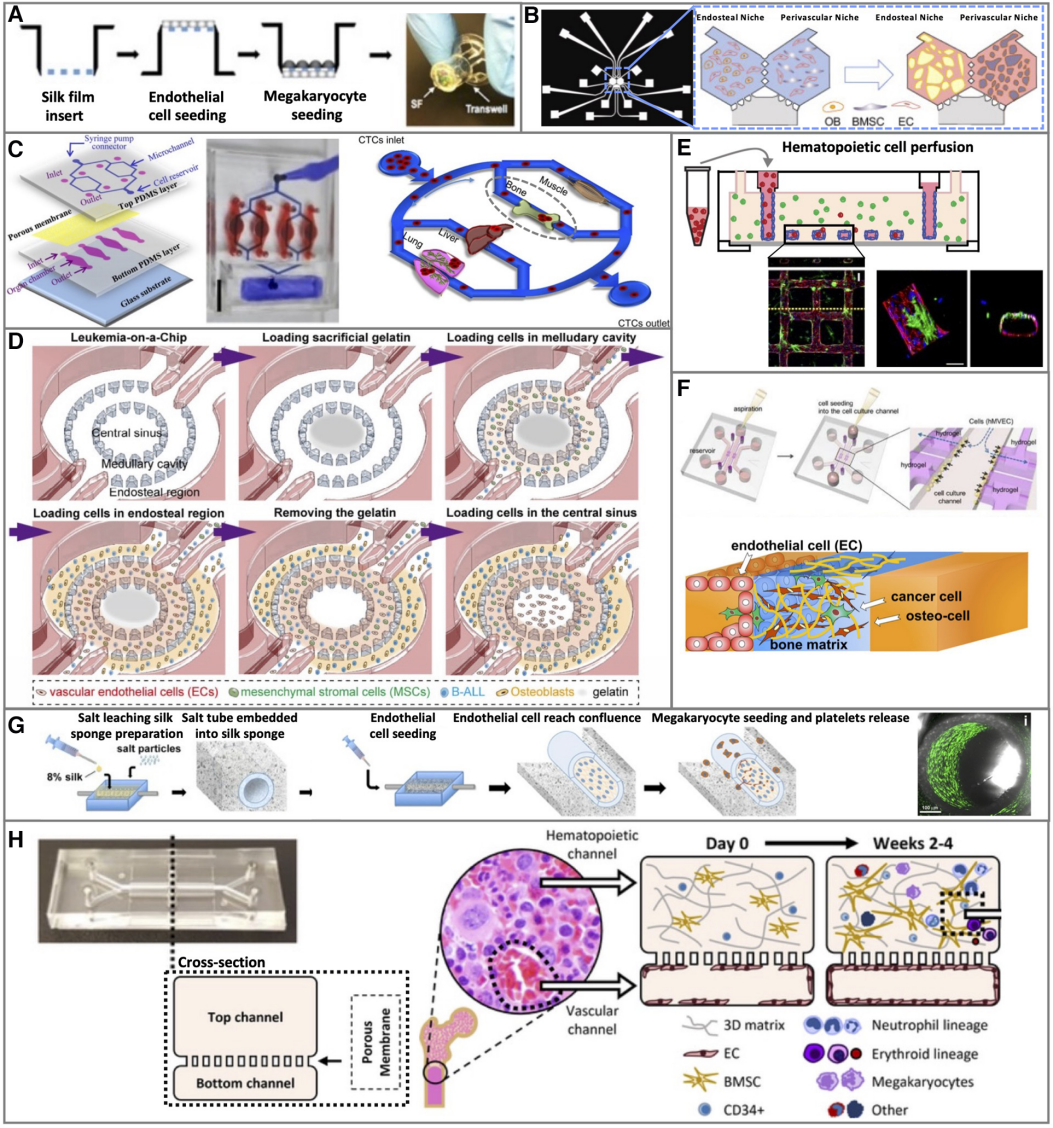
Representative 2D and 3D bone marrow vascular *in vitro* models. (**A**) 2D static, (**C**) 2D microfluidic, and (**B,D–G**) 3D BM vascular *in vitro* models. Figure adapted from: (**A**) Di Buduo et al.; Blood (105). (**B**) Glaser et al.; Biomaterials (130). (**C**) Kong et al.; Oncotarget (121). (**D**) Ma et al.; Science Advances (123). (**E**) Kotha et al.; Stem Cell Research & Therapy (134). (**F**) Bersini et al.; Biomaterials (131). (**G**) Di Buduo et al.; Blood (105). (**H**) Chou et al.; Nature Biomedical Engineering (119).

In another hybrid 2D model, murine primary BM cells were grown on collagen, then a porous membrane was laid on top, and sealed with a microfluidic channel layer. Here, HUVECs were seeded on a basement membrane extracts coating and grown to form a vessel wall-mimicking monolayer (121) (Figure 2C).

In Rafii's lab, a method to maintain long-term survival and facilitates organ-specific purification of primary ECs was generated, based on the expression of E4ORF1 in serum/cytokine-free conditions (122). E4ORF1-HUVECs were employed to develop the vascular niche of an engineered platform hosting other cells types: human leukemic and embryonal carcinomas cells (122), BM-derived LT-HSCs (101) or hCD34^+^ cord blood (CB) stem and progenitor cells (102).

A coculture of HUVECs with human primary HSPCs was performed in a 50 µm wide microwell allowing one EC and one HSPC to be dispensed per well (103).

HUVECs or primary endothelial colony-forming cells have been used for tri-cultures with primary human BM-MSCs and primary hematopoietic CB-derived CD133^+^ CD34^+^ cells (104).

An improved transwell system has been developed mounting common transwell with silk films, functionalized by surface coating or entrapment of ECM components (collagen, fibrinogen, fibronectin, or laminin). In this model, CB-derived primary ECs and MKs were seeded on opposite sides of the functionalized membrane (105) (Figure 2A).

A higher level of the biological complexity of the 2D BM vascular *in vitro* model was achieved by introducing the primary BMECs isolated from human BM aspirates. These cells were cultured as monolayers with primary HSPCs, both as direct coculture and in transwell (106).

### 4.2. 3D bone marrow *in vitro* models

The 2D systems are reproducible and easy to use, however, they have limitations in mimicking fundamental *in vivo* BM vascular features and complexities such as cell-cell or cell-matrix interactions, hampering the biological accuracy to model the BM vasculature and its surrounding niche. Recently, the development of 3D models and organ-on-a-chip fabrication allowed the recapitulation of the *in vivo* features of the vasculature more closely.

#### 4.2.1. Murine 3D bone marrow *in vitro* models

BM vascular 3D murine *in vitro* models include both hybrid (murine and human cells) and murine-derived cells (Tables 1, 2). Rat primary BM-MSCs in collagen gel were cocultured with primary rat aortic ECs. The ECs were either bound to dextran-coated Cytodex 3 microcarrier beads embedded in a 3D fibrin gel underneath to allow vascular sprouting (99), or cultured on the surface of collagen gel-BM-MSCs modules grown under shear stress (107). In this model, the empty spaces created by the collagen modules randomly packed were considered perfusable EC-lined channels (107).

**Table 1:** Bone marrow vascular *in vitro* models for cardiovascular-related research.

Species		*In vitro* model features					Cell type					Topic	Ref
Murine 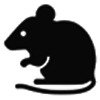	Human 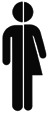	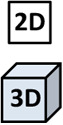	ECM	Static	Microfluidic	Shear stress	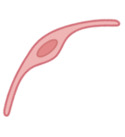 Endothelial	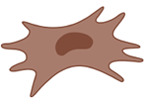 Mesenchymal stem/stromal	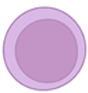 Hematopoietic/progenitor	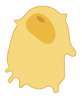 Osteoblasts	Monocytes/ Macrophages Adipocytes Fibroblasts 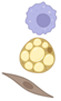	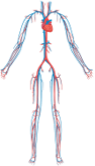	
CARDIOVASCULAR-RELATED													
**2D** BONE MARROW *in vitro* MODELS													
Rat		2D	–	√		**NO**	Primary aortic ECs	Primary BM-MSCs				Angiogenesis	(99)
Mouse & Human (hybrid)		2D	Coating	√		**NO**	HUVECs or HMVECs or HAECs Gelatin		Primary HSPCs (BM)			Mechanisms of ECs-HSPCs interaction	(100)
Mouse & Human (hybrid)		2D	–	√		**NO**	E4ORF1^+^ HUVECs		Murine primary LT-HSPCs (BM)			LT-HSCs expansion	(101)
Human		2D	–	√		**NO**	E4ORF1^+^ HUVECs		Primary HSPCs (cord blood)			HSPCs expansion	(102)
Human		2D	Coating	√		**NO**	HUVECs Fibronectin		Primary HSPCs (cord blood)			HSPC polarization	(103)
Human		2D	–	√		**NO**	HUVECs or ECFCs (cord blood)	Primary BM-MSCs	Primary HSPCs (cord blood)			HSPC expansion *in vitro*; cell-cell interaction	(104)
Human		2D	ECM-coated silk films	√		**NO**	EPCs (cord blood)				Primary MKs	Platelet production	(105)
Human		2D	Coating	√		**NO**	HUVECs or BMECs Gelatin	Primary BM-MSCs	Primary HSPCs (cord blood or peripheral blood)			HSPCs proliferation/differentiation	(106)
**3D** BONE MARROW *in vitro* MODELS													
***murine 3D*** *in vitro models*													
Rat		3D	FIBRIN COLLAGEN	√		**NO**	Primary aortic ECs	Primary BM-MSCs				Angiogenesis	(99)
Rat		3D	COLLAGEN		√	**YES**	Primary aortic ECs	Primary BM-MSCs				Cell-cell interaction	(107)
Mouse		3D	FIBRIN- COLLAGEN		√	**YES**	C166 cell line Yolk sack	Primary BM-MSCs	Primary HSPCs (BM)	Osteoblast-diff. primary BM-MSCs		Maintenance of HSPCs	(108)
Mouse		3D	Tissue explant		√	**NO**	Primary bone marrow cells (BMCs)					Model of BM damage	(109)
Mouse & human (hybrid)		3D	ALGINATE or MATRIGEL		√	**YES**	HUVECs Fibronectin				Primary MK (mouse) or hiPSC derived MKs	Platelet production	(110)
***human 3D*** *in vitro models*													
Human		3D	ALGINATE		√	DYNAMIC	HUVECs		Primary HSPCs (cord blood)	Osteo-diff Primary BM-MSCs		Maintenance of HSPCs, cell-cell interaction	(111)
Human		3D	ECM cell- produced	√		**NO**	HDMECs	Primary HSPCs (cord blood)	Primary HSPCs (cord blood)			Maintenance of HSCs	(112)
Human		2D	ALGINATE or MATRIGEL	√		**NO**	EPCs (cord blood)		Primary HSPCs (cord blood)	Osteoblast-diff primary BM-MSCs	Adipo-differentiated primary BM-MSCs	Manteinance of HSPCs	(113)
Human		2D	ECM coated silk films	√		**NO**	EPCs (cord blood)				CD61^+^ MKs	Manteinance of HSPCs	(114)
***human 3D self-assembled*** *vascular networks in vitro model*													
Human		3D	FIBRIN		√	**NO**	HUVECs	Primary BM-MSCs				Angiogenesis	(115)
Human		3D	FIBRIN		√	**NO**	HUVECs	Primary BM-MSCs				Angiogenesis	(116)
Human		3D	FIBRIN		√	**YES**	HUVECs	Primary BM-MSCs				Angiogenesis	(117)
Human		3D	FIBRIN-COLLAGEN		√	**NO**	HUVECs		Primary HSPCs (CORD BLOOD)	hFOB cell line	NHLF fibroblast cell line	HSPCs polarity	(103)
Human		3D	FIBRIN-COLLAGEN		√	**NO**	HUVECs	Primary BM-MSCs	Primary HSPCs (BM)	Osteoblast-diff. primary BM-MSCs		Manteinance of HSPCs; model of BM damage	(118)
***human 3D endothelial cell-lined*** *in vitro models*													
Human		3D	FIBRIN		√	**YES**	HUVECs Fibronectin Collagen	Primary BM-MSCs	Primary HSPCs (peripheral blood)			Model of BM damage and diseases	(119)
Human		3D	FIBRONECTIN-COLLAGEN-LAMININ		√	**YES**	EPCs (cord blood) or HMECV-d				Primary MKs	Platelet generation	(105)

**Table 2:** Bone marrow vascular *in vitro* models for cancer research.

Species		*In vitro* model features					Cell type					Disease	Ref
Murine 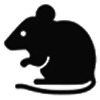	Human 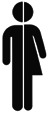	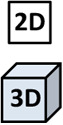	ECM	Static	Microfluidic	Shear stress	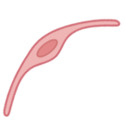 Endothelial	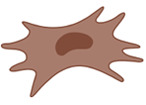 Mesenchymal stem/stromal	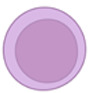 Hematopoietic/progenitor	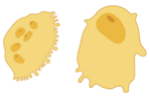 Osteoclasts/Osteoblasts	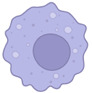 Monocytes/macrophages	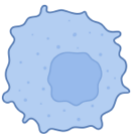 Cancer	
Cancer													
**2D** Bone MARROW *in vitro* MODELS													
Mouse & Human (hybrid)		2D	–		√	**NO**	HUVECs					Leukemia (AML)	(120)
Rat & Human (hybrid)		2D	Coating		√	**YES**	HUVECs Cultrex	Primary BM cells Collagen	Primary bone marrow cells (BMCs) Collagen			Breast triple neg	(121)
Human		2D	–		√	**NO**	E4ORF1+ HUVECs					Lukemia (APL) and embryonic carcinoma	(122)
**3D** BONE MARROW *in vitro* MODELS													
***murine 3D*** *in vitro models*													
Mouse		3D	FIBRIN		√	**NO**	C166 cell line (yolk sack)	OP9 cell line (BM)				Leukemia (ALL)	(123)
Mouse & human (hybrid)		3D	FIBRIN		√	**YES**	HUVECs	Primary BM-MSCs		Osteoblast-differentiated BM-MSCs	RAW264.7 cell line (macrophages)	Brest triple negative	(124)
***human 3D*** *in vitro models*													
Human		3D	FIBRIN	√		**NO**	HUVECs	Primary BM-MSCs				Multiple myeloma	(125)
Human		3D	COLLAGEN-MATRIGEL	√		**NO**	iPSC-derived ECs	iPSC-derived MSCs	iPSC-derived HSPCs			Myelofibrosis, multiple myeloma, leukemia (ALL)	(126)
Human		3D	FIBRIN		√	**NO**	HUVECs	Primary BM-MSCs		hFOB 1.19 cell line (osteoblasts)		Leukemia (ALL)	(123)
***human 3D self-assembled*** *vascular networks* *in vitro models*													
Human		3D	StarPEG- HEPARIN HYDROGEL	√		**NO**	HUVECs	Primary BM-MSCs				Leukemia (AML)	(127)
Human		3D	BONE MATRIX		√	**YES**	HUVECs	Primary BM-MSCs				Breast triple neg	(128)
Human		3D	COLLAGEN		√	**NO**	HUVECs	HS5 cell line (BM)				Leukemia (AML-CML)	(129)
Human		3D	FIBRIN		√	**NO**	ECFCs (cord blood)	Primary BM-MSCs	Primary HSPCs CD34^+^ (cord blood)	hFOB 1.19 osteoblast cell line		Breast triple neg	(130)
***human 3D endothelial cell-lined*** *in vitro models*													
Human		3D	COLLAGEN		√	**NO**	HUVECs Matrigel			Osteoblast- differentiatedBM-MSCs		Breast triple neg	(131)
Human		3D	FIBRIN and COLLAGEN		√	**YES**	HUVECs Collagen and Fibronectin		Primary HSPCs CD34^+^ (cord blood)			Cancer drugs	(132)
Human		3D	BONE MATRIX		√	**YES**	HUVECs Fibronectin	Primary BM-MSCs		Osteoblasts differentiated BM-MSCs Osteoclasts differentiated monocytes		Cancer drugs	(133)
Human		3D	COLLAGEN		√	**YES**	HUVECs	HS5 or HS27a cell lines (BM)			Primary monocytes (peripheral blood)	Leukemia (AML)	(134)
Human		3D	HYALURONIC ACID-GELATIN		√	**NO**	Primary BMAECs and BMSECs	Primary BM-MSCs		Osteoblast- differentiated primary BM-MSCs		Leukemia (AML)	(135)

Murine tri-culture has been realized by coculturing mouse ECs from the yolk sack (C166 cells) with BM-MSCs and leukemic B-ALL cells in a 3D fibrin hydrogel, in the two inner regions of a 3 concentric rings microfluidic chip. The niche cells, BM-MSCs, and ECs were placed in the middle ring area, while the B-ALL cells were in the central region (123).

A murine tetra-culture was generated in a microfluidic chip with two channels divided by a semi-porous membrane to physically separate the vascular and the BM niche components. The ECs C166 were grown as a monolayer on the lower side of the membrane under fluidic conditions (30 µl/h). On the apical side, the BM niche composed of primary BM HSPCs or whole BM cells and primary BM-MSCs in a fibrin-collagen gel was topped with a layer of BM-MSCs-derived osteoblasts (108).

An engineered murine BM vascular *in vitro* model was generated directly *in vivo*, implanting subcutaneously a polydimethylsiloxane (PDMS) device with a central cylindrical cavity functioning as a scaffold, resulting in a bone-like cylindrical disk tissue with a central region of blood-filled marrow. This engineered BM was surgically removed 8 weeks after implantation, punctured in multiple places to allow access to the culture medium, and then cultured in a 5 layers PDMS microfluidic device. Here, a compatible central cylindrical chamber to accommodate the engineered BM was encapsulated in a sandwich-like structure with two porous membranes and microfluidic channels (109).

One of the most important vascular features consists in the hemodynamic forces applied to the vascular walls formed by ECs, therefore advanced vascular models require shear stress to mimic *in vivo* blood flow. Two hybrid human-murine 3D microfluidic BM *in vitro* models that include shear stress on the endothelium were generated. The first is a bioreactor-on-a-chip composed of an upper and a lower microfluidic channel separated by a series of pillars (2 µm apart). The upper channel was injected with a 3D Matrigel or alginate gel embedding murine primary MKs, and a monolayer of primary HUVECs was cultured on fibronectin in the lower channel to mimic the BM vessel wall. In this model, the endothelial monolayer was directly exposed to a controlled flow (12.5 µl/h) by syringe pumps (110). In the second one, a 3-channel microfluidic model developed by Jeon and colleagues a flow of 2 μl/min was introduced via the lateral channel to condition the central channel vasculature network flow resulting in wall shear stress of 0.25 dynes/cm^2^ (124).

#### 4.2.2. Human 3D bone marrow *in vitro* models

To increase the complexity of the 3D BM vascular models, a great effort was put into isolating and/or employing human-derived cells both in static and microfluidic conditions (Tables 1, 2). These models mainly combined non-BM-derived primary ECs with BM stromal cells by culturing HUVECs or a few different primary ECs such as ECs from CB or derma as an endothelial component. Therefore, the human BM niche-stromal components were the real BM tissue-specific cells in these models.

A tri-culture of HUVECs mixed with primary CD138^−^ BM-MSCs and patient-derived cancer cells was performed in a fibrinogen scaffold generated from patient BM-derived fibrinogen, naturally found in the plasma of BM supernatant (125).

HUVECs-MSCs coculture has been also used to generate a 3D static and dynamic tetra-culture with osteoblasts differentiated from primary umbilical cord-derived MSCs, and primary CB-derived CD34^+^ HSPCs. Oliveira et al. generated a BM in liquefied capsules, to facilitate cell movement and self-organization, following three main sequential steps: (i) engineering the endosteal niche by seeding human MSCs with surface-functionalized microparticles, (ii) bioencapsulation of the endosteal niche with HUVECs and HSPCs in alginate, and (iii) production of a multilayered membrane layer-by-layer followed by core liquefaction (111). Another 3D BM static model employed spheres was, generated by the magnetic levitation method, to avoid the use of scaffolds or exogenous matrices and to allow the cells to form the extracellular matrix (108).

Primary ECs from derma, primary BM-MSCs, and primary CD34^+^ HSPCs from CB were combined in a tri-culture, assembled in the 3D Bio AssemblerTM System that mixed all cells (112).

A static penta-culture 3D BM model was realized by embedding in Matrigel or alginate hydrogel primary cells: CB-derived HSPCs and EPCs, BM-MSCs, osteoblasts, and BM-MSCs-derived adipocytes (113).

The outstanding research of Palikuqi et al. identified a new method to obtain a durable hemodynamic, self-organizing, large-volume 3D vascular system in a Matrigel-free matrix, composed of a mixture of laminin, entactin, and type-IV collagen (LEC matrix). It is based on the “reset” of vascular endothelial cells (R-VECs) through the transient reactivation of the embryonic-restricted ETS variant transcription factor 2 (ETV2). These findings could lead to a revolution in the organoid field thanks to the ability to vascularize organoids and tumoroids (136). In the context of *in vitro* BM modeling, it would open the possibility of recreating the vascular niche in a complex 3D environment for pharmaceutical studies but also offer the opportunity to develop functional and perfused implantable tissues ex vivo.

A vascularized BM organoid based on human induced pluripotent stem cells (iPSCs) was developed in a hydrogel composed of collagen I and IV, and Matrigel. The used medium was supplemented with vascular endothelial growth factor C and the characterization reveals the presence of iPSC-derived ECs, MSC, HSPCs, myelomonocytic, megakaryocytes and immature erythrocytes with human BM cells characteristics. Moreover, this human BM organoid were engrafted with different types of cells to mimic human diseases; specifically: iPSC-derived HSPCs derived from patients with myelofibrosis or healthy individuals, primary cancer cells from patients affected by multiple myeloma, acute lymphoblastic leukemia (ALL), and myeloid leukemias (126).

The aforementioned ring shape microfluidic chip (123), used to culture murine cells, was also employed in an all-human fibrin hydrogel-based 3D model. In this case, HUVECs were seeded in the central sinus region, a mixture of HUVECs, primary BM-MSCs, and leukemia cancer cells in the inner ring, and a mixture of osteoblasts (hFOB cells) and leukemic cancer cells leukemia in the outer ring channel (123) (Figure 2D).

A 3D hydroxyapatite porous scaffold placed in, and, connected to a perfusion bioreactor was used to coculture adipose tissue-derived stromal vascular fraction, which includes primary mature ECs, endothelial progenitor cells, pericytes, and MSCs, with osteo-differentiated primary BM-MSCs. This engineered vascularized BM niche was perfused at the rate of 300 μl/min with a suspension of CD34^+^ CB-HSPCs (114).

#### 4.2.3. Human 3D bone marrow self-assembled vascular networks *in vitro* models

To mimic the *in vivo* vascular structure, 3D human BM *in vitro* models were developed based on self-assembled vascular networks (Tables 1, 2). HUVECs have been cocultured in tri-cultures with primary BM-MSCs and cancer cells in a hydrogel drop of approximately 200–300 Pa stiffness, optimal for the development of a robust endothelial network (127).

Microfluidic human BM 3D *in vitro* models are characterized by controlled and/or induced flow for nutrient exchange in all-human cell cultures. 3D vascular models made of capillary-like structure networks were formed by HUVECs cocultured with primary BM-MSCs. The mesenchymal cells adopted pericyte-like localizations among the network system, both in a 3D decellularized bone matrix (128) and in fibrin matrixes within 3D microfluidic chips with different designs and geometries (115–117).

Maturano-Kruik et al. developed a capillary-like structures network model seeding the ECs and MSCs within a 3D decellularized bone tissue's trabecular scaffold that after a maintenance phase, was placed in the microfluidic chip and exposed to a controlled flow (128).

The microfluidic chips of Jeon et al. (115) and Mykuliak et al. (117) were designed with a central gel channel, for HUVEC-MSC coculture, between two lateral channels for culture medium, separated by pillars. In this last HUVEC-MSC coculture model, the shear stress on ECs was exerted by interstitial flow and it was estimated to be 0.0065 Pa (117).

Carrion et al. used a 3D penta-channel microfluidic chip with three central parallel gel channels and two lateral channels for medium supply. The central gel chamber was filled with fibrin gel only, and the two adjacent channels with fibrin-embedded ECs and MSCs respectively (116).

Capillary-like structures were also obtained by culturing HUVECs in a 3D gel in the absence of perivascular MSCs (103, 129). In one case, cells were cultured in a microfluidic chip with three parallel microchannels, partitioned by trapezoid-shaped pillars. The central channel was filled with collagen gel, and one lateral channel with ECs monolayer, grown on the pillar-collagen interface of the central channel to provide the space for angiogenic sprouting and neo-vessel formation. The other lateral channel was seeded with leukemia cancer cells followed by injection in the channel reservoirs of BM-MSCs (HS5 cells) in collagen gel (129).

In a second work, the microfluidic chip was composed of two lateral media channels and three main parallel gel channels (103). The two lateral channels mimic the BM vascular niche and the endosteal niche, hosting the vascular network generated by HUVECs and hFOB osteoblasts in collagen fibrin gel, respectively. The central channel was loaded with primary HSPCs in collagen fibrin gel. Another lateral channel, separated by the medium channel from the vascular compartment, holds mitomycin C-treated fibroblasts (NHLF cells) and was used as a cytokine-secreting compartment (103).

The BM perivascular and endosteal niches were also simultaneously reproduced in a microfluidic device with two hexagonal chambers connected by three symmetric two-way ports that allow nutrient diffusion and cell migration. Primary CB-derived ECs were cultured in both chambers in fibrin hydrogel to form a vascular network, mixed either with hFOB 1.19 osteoblasts to form the endosteal niche or primary BM-MSCs to form the perivascular niche on-a-chip (130) (Figure 2B).

The biological complexity of a 3D BM *in vitro* model can be enhanced by adding other cell types in culture, to better mimic the tissue microenvironment. Other models characterized by the formation of a BM vascular network and its niche were realized in 3D microfluidic devices with parallel 3- or 5-channels. In the central channel, HUVECs were cultured with primary BM-MSCs, osteo-differentiated primary BM-MSCs, and primary HSPCs (118) or with primary BM-MSCs and macrophages (115) in ECM gel. Hence, this type of model allows the mixing of the vascular and the endosteal niches' cellular components. In the central channel of the 3-channel microfluidics device, a microvascular network with highly branched structures was grown in 3D fibrin, supported by medium from the two lateral channels (115). In the central channel of the 5-channel microfluidic system BM-M-SCs were cultured on a mix of collagen and dopamine-HCl to differentiate in osteoblasts as a monolayer, then a fibrin-collagen hydrogel with a mixture of endothelial, mesenchymal and hematopoietic cells was injected on top of the osteoblasts to obtain a 3D human BM-on-a-chip. The two side channels serve for medium supply, and the two external channels were filled with BM-MSCs in fibrin-collagen hydrogel to provide further support to the BM niche (118).

#### 4.2.4. Human 3D bone marrow endothelial cell-lined *in vitro* models

To mimic the vascular geometry and endothelial distribution *in vivo*, 3D vascular microfluidic models were designed to grow ECs on the channel walls to generate an endothelial monolayer creating a lumen.

A microfluidic device with three parallel central channels and two lateral channels for medium supply was designed to create a lumen (137). In this device, a HUVEC monolayer was cultured on Matrigel in the central channel to form a vascular wall-like geometry lumen with a squared section where medium and circulating cells were flown trought (131). In the side channel, in contact with the endothelial central channel, osteo-differentiated primary BM-MSCs were cultured in 3D collagen to generate an osteo-conditioned microenvironment (131) (Figure 2F).

A 3D 1-channel BM microfluidic model, part of a body-on-a-chip, consists of primary HSPCs embedded in a 3D fibrin gel and a HUVECs monolayer placed on opposite site of a porous membrane. Here the endothelial monolayer was directly exposed to controlled shear stress (0.2 dynes/cm^2^) (132).

A similar microfluidic model included also the primary BM-MSCs with primary HSPCs embedded in fibrin in the top compartment, and a HUVEC monolayer grown under shear stress (1.2–1.6 μl/min) as a vascular compartment in the bottom (119) (Figure 2H).

The BM endosteal niche was partially recapitulated in a 3D microfluidic chip, as part of a vascularized multi-organ tissue system (133). Here, the bone tissue was obtained by coculturing both BM-MSCs differentiated osteoblasts and primary CD14^+^ monocytes-derived osteoclasts within a decellularized bone scaffold on top. To mimic blood vessel walls, the porous membrane was seeded with pericytes-mimicking BM-MSCs on the upper surface and with HUVECs mixed to a small fraction of BM-MSCs on the lower side grown under shear stress (1.88 dynes/cm^2^ max) (133).

A 3D microfluidic vascular network that mimics the vasculature geometry was created by growing HUVECs on a collagen gel grid characterized by a series of squared blocks design, to allow the formation of a rounded-shaped monolayer under gravity-mediated flow. In the collagen gel, the BM components were embedded as primary BM-MSC or BM fibroblasts cell lines (134) (Figure 2E).

A tubular-shaped vascular microfluidic device made with a silk microtube surrounded by a porous silk sponge was seeded with primary CB- or derma-derived ECs in the silk microtube lumen and exposed to shear stress. Then, primary HSPCs-derived human MKs were seeded into the silk sponge (105) (Figure 2G).

Finally, the 3D BM vascular *in vitro* model, which better mimics the *in vivo* BM vasculature was generated using organ-specific primary BMECs, isolated via magnetic separation from frozen human BM-MNCs. Arterial and sinusoidal ECs were isolated via double selection for CD146^+^/NG2^+^ or CD146^+^/NG2^lo/−^, respectively (135). These cells were grown to replicate the major 3D BM niches in a deconstructed way in a microfluidic chip designed with 4 non-communicating parallel channels that can be exposed to control flow, where the four different niche cell populations were encapsulated in ECM-derived hydrogel: (I) arteriolar BMECs, (II) sinusoidal BMECs, (III) primary BM-MSCs and (IV) osteoblasts derived from osteo-differentiated primary BM-MSCs (135).

In the next sections, we describe how the reported models have been employed for either cardiovascular or cancer research.

### 4.3. Bone marrow vascular models used for cardiovascular research

Modeling the BM vasculature is essential to study inside-out cell mobilization and for the comprehension of mechanisms regulating this process important in both cancer and cardiovascular research. Many laboratories have focused on developing *in vitro* models of the BM vasculature that can be used for cardiovascular studies. These, range from cultures that allow HSPCs maintenance *in vitro* to study hematopoiesis (108, 109, 111–113, 118, 119), to devices to investigate cell-cell interaction (103, 107, 114, 115) and vasculogenesis (99, 115–117). Advanced systems recapitulate key features of BM damage (109, 118, 119) or diseases (119). Other interesting research has developed devices for platelet production *in vitro* that can be employed for clinical purposes (105, 110).

Two different approaches, involving mouse primary cell growth in a BM-rich microenvironment, were able to maintain the proportions and characteristics of HSPCs for up to 7 and 14 days, respectively (108, 109). However, to finely study human hematopoiesis human cells are required (138, 139). Two unconventional methods were adopted to generate spherical-shaped human BM cocultures (111, 112). Organotypic multicellular spheres developed through magnetic levitation in the absence of scaffold and exogenous matrices stimulate cells to produce their extracellular matrix. This work opens the door to the realization of more complex organoids applicable in the field of regenerative medicine (112). The cellular release of ECM components and HSPCs supportive factors was observed also in the other spherical-shaped culture, the “human bone marrow–in–a–liquefied-capsule” when placed under dynamic fluid (111).

Braham et al., developed a fully primary human cell 3D coculture useful to understand the interaction of HSPCs with other BM cells and the ECM in normoxic, hypoxic, and hyperoxic conditions. They show that Matrigel is a better substrate for coculturing HSPCs with osteo-vascular components compared to alginate (113). The interaction of HSPCs with the BM microenvironment is crucial for *in vitro* long-term culture maintenance and in deciding their fate (140). A human 3D microfluidic device was able to recapitulate the *in vivo* observed HSPCs polarization (141) proving to be a great model to study HSPC polarization mechanisms, which are still unknown (103).

Butler et al., demonstrate the fundamental role of ECs in cells expansion, in the E4ORF1-ECs based vascular niche engineered platform ECs resulted to enhance proliferation and maintencance of human leukemic and embryonal carcinomas cells (122), BM-derived LT-HSCs (101) and cord blood-derived hCD34^+^ stem and progenitor cells (102) in serum/cytokine-free conditions.

Born and colleagues demonstrate that a self-assembled vascular structure not only maintains the HSPCs in their undifferentiated state but also preserved the osteogenic potential of BM-MSCs (114). The influence of ECs on BM-MSCs was evaluated also in a rat-based 3D dynamic system demonstrating that the combination of shear stress with the vascular component induces BM-MSC differentiation in α-SMA^+^ pericytes and stimulates their migration through ECs (107). This model is of great interest as the presence of BM-perivascular cells is fundamental to mimicking BM vasculature. A similar study conducted with human primary ECs, and BM-MSCs, cultured in 3D microfluidic condition (115) confirms the transition of MSCs toward α-SMA^+^ mural cells when cocultured with ECs and that this transition is further enhanced by the presence of angiogenic factors such as angiopoietin-1 and TGF-1β. Interestingly, the α-SMA^+^ mural cells colocalize with ECs suggesting a contact-dependent mechanism to induce BM-MSCs differentiation. These last studies prove the important support of ECs on BM-MSCs fate.

Other research has instead focused on the influence of BM-MSCs on ECs and the vasculogenesis process. Jeon et al., demonstrate that the vascular component developed a higher number of network branches and display a reduced vessel diameter in the presence of BM-MSCs (115). Rat BM-MSCs embedded in a 3D microenvironment enhanced sprouting and proliferation of ECs through paracrine signals (99). A human-based microfluidic chip with cells embedded in fibrin gel confirmed the capability of BM-MSCs to sustain ECs vascular network formation. Moreover, they have shown that BM-MSCs promote basement membrane deposition by ECs and that the perivascular localization of BM-MSCs occurs through the interaction of their integrin adhesion receptor α6β1 and basement membrane laminin (116). Analyzing the fibroblast effects on EC microvasculature they observed that fibroblasts enhance vascular network formation even faster than BM-MSCs (116). The interaction of ECs with MSCs derived from BM or adipose tissue was compared in a human cell-based 3D microfluidic model. Both types of MSCs support network formation, but the BM-MSCs resulted in a more organized, interconnected, and denser ECs network and in higher expression of genes characteristic of pericyte and ECs compared to the coculture of ECs with adipose-derived MSCs (117).

To develop effective drugs to recover BM damage or treat BM diseases it is important to mimic the BM alteration characteristics of the pathology. The mouse cell-based model of Torisawa et al. faithfully recapitulates BM damage observed in live irradiated mice and the BM recovery after administration of G-CSF, a largely used drug to treat victims of radiation accidents (109, 142). In a 3D human osteovascular niche on-a-chip the endosteal component demonstrates a protective activity on HSPCs reducing their apoptosis following ionizing radiation exposure (118).

Chou and colleagues developed an advanced human microfluidic BM-on-a-chip suitable to study drug and irradiation toxicity as well as BM recovery. In this chip, able to maintain myeloerythroid proliferation and differentiation, under dynamic condition was recapitulated the reduction in neutrophils and total cell count induced by the chemotherapeutic agent 5-fluorouracil; and also neutrophils and erythroid cytotoxicity induced by AZD2811, a potential cancer drug currently in phase II of clinical development (119). The hypothesis that AZD2811 selectively targets dividing neutrophil and erythroid precursors was assessed inside this microfluidic chip. Moreover, the observed differences in cytotoxicity between low and high levels of γ-radiation resemble human radiation sensitivity. Interestingly, the damaged cells can recover inside the model, an important characteristic for pharmaceutical research (119).

Finally, HSPCs derived from Shwachman–Diamond syndrome (SDS) patients were cocultured with normal BM-MSCs and ECs. SDS is a rare genetic disorder resulting in bone marrow failure, characterized by neutropenia and other BM-cell defects. The SDS-on-a-chip faithfully reproduces the disease, opening new frontiers for translational, drug development, and cytotoxicity studies (119).

One of the problems of the clinical practice that is trying to be solved by these new *in vitro* technologies is represented by platelet transfusion. Nowadays, all transfusion platelets are obtained by human donors and can cause immunogenic reactions in the receiving patient. Two laboratories realized two different bioreactors able to reproduce thrombopoiesis, in both cases the presence of vascular shear stress enhanced platelet collection (105, 110). Thon et al., utilized human induced pluripotent stem cells (iPSCs)-derived MKs that could potentially produce an unlimited number of functional human platelets and overcome the immunogenicity problem (110). The model of Buduo et al., is of great interest because they designed a functionalized silk-based BM system that could replace the endothelial layer, required to promote thrombopoiesis *in vitro*, facilitating the scaling-up for future massive platelet production (105).

### 4.4. Bone marrow vascular models used for cancer research

The BM is the semi-solid tissue where hematologic cancers such as leukemia and myelomas originate (143, 144). The BM is also one of the most common sites for solid tumor metastasis as breast and prostate cancer (145) (Figure 1). 2D and 3D BM *in vitro* vascular models have been employed to study both liquid or hematologic cancers and solid cancer metastasis.

#### 4.4.1. Hematologic cancers

Leukaemia and myeloma begin during hematopoiesis with cell genetic alterations. Most leukaemia involves white blood cells, and some affect other types of blood cells. When these cells over over-proliferate, they crowd out normal BM cells with consequent hematopoiesis alterations leading to the development of the disease. Leukemic cells leave the bone marrow niche through the BM vasculature to enter the bloodstream, where they can spread to other organs. Leukaemia is classified based on the speed of progression, that which could be acute or chronic, and on the type of blood cell involved, myeloid or lymphoid (146).

Acute leukaemias (AL) are characterized by immature blood cells with high intrinsic proliferative potential that leads to a fast progression of the disease. Altered immature blood cells with a myeloid commitment, cells able to differentiate into MKs, red or non-lymphocyte white blood cells during hematopoiesis in health, cause acute myeloid leukemia (AML), which is the most common type of acute leukemia in adults (147).

In leukemic patients it has been observed that an increased BM vascularization is a key feature, however, this event has not been largely studied for solid tumor progression and metastasis (148). Angiogenesis was studied with a 3D BM microfluidic chip showing that ECs (HUVECs) sprouting and neo-vessel formation were increased by the presence of AML cells (HL60 cells) compared to control (EC medium). In the presence of HS5BM-SCs, to mimic the BM niche, angiogenesis was more pronounced, and, in coculture with AML cells, it was further enhanced, but characterized by a larger number of isolated endothelial tips without multicellular stalks, reflecting what was found in AML patient biopsies. ECs migrated for longer distances in the collagen matrix, with a higher number of endothelial tips but lower vascularization were found using chronic myeloid leukemia (CML) cells (K562 cells) in the absence of stromal cells (129). CML originates from partially mature myeloid white blood cells and is characterized by slower progression compared to AML. In turn, ECs play an important role in CML. Coculture of HUVECs with a murine AML cell line in a 2D model showed that ECs support the expansion of rare leukemic cells, known as LICs representing a subset of self-renewing cells able to generate an aggressive AML (120). This was also shown in the 3D BM vascular model developed by Bray et al., where both AML cell lines (KG1a, MOLM13, MV4-11, OCI-AML3) and primary patient-derived AML cells exerted preference to adhere and proliferate along the endothelial network (HUVECs-BM-SCs), further highlighting the importance of the interaction between AML cells and the vascular niche. In accordance with these findings, this model enabled further research on drug resistance, one of the main challenges in clinical treatment. It has been observed that the 3D tri-culture model was more resistant to two antineoplastic drugs commonly used to treat AML compared to simpler 2D and 3D mono-cultures (AML cells only) (127). In a perfusable 3D microfluidic vascular model with tubular HUVECs vessels cocultured with stromal BM fibroblast cell lines (HS27a or HS5), patient-derived primary AML cells adhesion and extravasation were tested under shear stress in combination with primary monocytes. Interestingly, AML cells did not exhibit preferential adhesion or extravasation in the presence of either stromal cell type, in contrast, leukemic cells without monocytes adhered and extravasated more to and across vessels cocultured with HS27a when compared to HS5 cells. These findings underline the importance of complex 3D models that mimic *in vivo* multicellular components (134) (Figure 2E).

To explore the AML cells-BM niche components specific interaction, a deconstructed microfluidic BM niches on-a-chip (NOC) with arterial (BMAECs), mesenchymal (BM-SCs), sinusoidal (BMSECs) and osteoblastic niches was perfused with AML cells (MOLM13). AML cells were preferentially lodged within the osteoblastic and the arterial niches rather than into the mesenchymal or sinusoidal niches (135). Acute lymphoblastic leukaemia (ALL) is another form of acute leukaemia and the most common cancer in childhood, characterized by the overproduction of immature and dysfunctional lymphoblast in the BM (149). A ring-shaped leukaemia-on-a-chip 3D BM model (Figure 2D) that resembles the *in vivo* spatial architecture and cellular composition of the leukaemia BM tissue was developed and adopted to study the heterogeneity in B-ALL human BM microenvironments based on their chemo resistance. It has been found that Ph^+^ SUP B-ALL cells cocultured with BM niche cells in the biomimetic device were more resistant to conventional chemotherapic than REH B-ALL, consistent with the insensitivity of Ph^+^ B-ALL to these types of drugs in the clinic. Furthermore, the leukemia-on-a-chip 3D BM model was designed to rescue the niche cells for downstream assay like single-cell RNAseq, a very powerful tool to characterize the BM microenvironment with limited cell input number. Finally, using a simpler ring-shaped murine 3D BM model with ECs and BM-MSCs it was observed that B-ALL cells attracted ECs. Niche cells promoted B-ALL cell cluster formation and reduced their motility over time (123).

The second most common hematologic malignancy is the MM, where clonal terminally differentiated B lymphocytes undergo abnormal proliferation followed by accumulation in the BM crowding out healthy blood cells. In MM abnormal monoclonal antibodies, paraproteins, are produced causing overly thick blood and kidney issues (150). To investigate the pathophysiology of MM, a 3D tissue-engineered BM (3DTEBM) derived from MM patients-BM supernatant that incorporated the BM components MM (MM1s, H929, RPMI8226 or primary), stromal, and ECs was established. It was observed that the presence of both stromal and ECs increased MM cell proliferation compared to single culture or coculture using other commercially available 3D systems, thanks to the 3DTEBM scaffold with fibrinogen cross-linking (125).

The classical feature of BM myelofibrosis consist in the deposition of reticulin and collagen fibers by marrow stroma (151) and the iPSC-based organoid model was able to recapitulate them when the donor cells derived from patients with myelofibrosis but not when derived from healty donor: increased soluble TGFβ levels, collagen 1 and α-SMA and reduced vascularization was observed (126).

#### 4.4.2. Solid cancer

Solid tumors such as breast cancer can spread from the primary site intravasating in the bloodstream to form secondary tumors at distant sites called metastasis. Metastasis is a multistep process where circulating tumors cells (CTCs) interact with ECs, forming the microvascular vessel wall, adhering and extravasate extravasating into the target organ to form metastasis (Figure 1). These key events of metastasis formation take place under hemodynamic shear stress exerted on ECs by blood flow (152). Triple-negative breast cancer (negative for oestrogen receptor, progesterone receptor, and HER2) is a highly invasive type of breast cancer, resistant to both hormonal therapy and treatments against HER2 (Trastuzumab), that develops metastasis in the bone with high frequency (153). Numerous BM vascular models *in vitro* were developed to study metastasis formation by human triple triple-negative breast cancer cells (MDA-MB-231) (121, 124, 128, 130, 131). It has been shown that CTCs firm adhesion to ECs (HUVECs) under shear stress was higher when HUVECs were cocultured with BM niche specialized cells compared to HUVECs cocultured with muscle cells, resulting in cancer cells organotropism for bone vasculature (121) (Figure 2C). In a model of self-assembled capillary-like network (HUVECs), cancer cell extravasation in the matrix increased in the presence of BM cells (BM-MSCs and osteoblasts). In contrast, it was observed a decreased extravasation in a shear stress-preconditioned vascular network or in the presence of inflammatory macrophages (inflammation model) within the BM niche (124). The extravasation step was further investigated in a microfluidic model with HUVECs forming a vascular lumen. MDA-MB-231 cells transmigrated across the HUVECs monolayer into the osteo-differentiated BM-MSC gel and travelled within the matrix more compared to the control. Extravasated cancer cells were able to proliferate and form micrometastasis of various sizes, ranging from 4 cells to 60 cells or more (131). A 3D BM vascular model was employed to investigate cancer cell migration to BM-specific niches. An increased MDA-MB-231 cell migration to the BM niches compared to the control was observed, without differences between perivascular and endosteal niches. The migrated MDA-MB-231 cells expressed more Ki-67, a proliferation marker, than non-migrated cells, consistent with high Ki-67 expression in bone lesions (130) (Figure 2B). All these 3D BM vascular models showed how the BM niche is important for the breast cancer cell metastatic process and enabled to highlight the cancer cell organotropicity. Finally, a 3D model with a bone matrix scaffold, to maintain the ECs viability and branching without the need of for specialized growth-promoting conditions culture medium, was developed to study metastatic colonization of MDA-MB-231 cells under static and interstitial flow conditions. It was reported that the interstitial flow promoted vascular branching and prevented cancer cell growth rate within the niche (128). The BM vasculature-on-a-chip models were also employed for drug toxicity studies. Two models were realized as part of a body on a chip (132, 133) to test the uptake and side effects of common chemotherapeutic drugs given to treat several cancers such as cisplatin and doxorubicin. The perfusable vasculatures were challenged in the luminal side when in contact with the apical bone marrow niche. Cisplatin was flowed through the vascular channel causing myeloid toxicity in the HSPCs BM niche, reducing the total number of cells in the HSPCs BM niche, in particular neutrophils and erythrocytes, recapitulating the known side effects of cisplatin in patients (neutropenia and anaemia) *in vitro* (132). In a vascularized bone model which includes a lot of components in common with the BM endosteal niche, osteoblasts were found to be more sensitive than osteoclasts when exposed to doxorubicin, as observed in pre-clinical studies, and, as expected, the endothelium showed decreased resistance (133).

## 5. Conclusions and future directions

Despite animal models, such as the broadly used murine models, are a powerful tool for genetic and physiological studies, they do not always mirror human biology and human medical disease conditions. About 8% is the rate of successfully translation of drugs from animal testing to human treatments (20). Thus, there is an unmet urgent need to develop physiologically relevant humanized models for hematopoietic and cardiovascular disorders. *In vitro* modeling has become a major focus of research in the field of vascular and cancer research in recent years, as scientists strive to understand the cellular and molecular mechanisms in human cells essential for the development of effective preventive and treatment strategies without the need for animal or human testing. Moreover, the advent of new strategies that enable a punctual characterization of the BM can not be ignored. Single-cell RNA sequencing has been used to identify and describe the characteristics of BM cells deriving from human and mouse (154–156) providing an exceptional tool for the validation of *in vitro* models exspecially in the context of disease modeling.

*In vitro* models can be used to simulate hemodynamic shear stress, an important component in cellular trafficking, providing insight into the disease mechanisms and how these might be prevented or treated. Furthermore, *in vitro* models can be used to investigate the effects of drugs or other treatments on the pathophysiology of specific organs. In this review, we summarized both murine and human 2D and 3D bone marrow vascular *in vitro* models with their cellular composition and experimental function that may be suitable to study *in vitro* cellular mobilization and homing in the BM via its vasculature, a central process in cardiovascular diseases and cancer. From this compilation emerged that these *in vitro* systems aimed to mimic at best the complexity of the BM environment. However, behind including ECs as vascular components, other BM cell types are required to establish a more *in vivo*-like and reliable model as different cell types release important factors orchestrating cellular fate, as explained in the first paragraphs of this review. Moreover, to study human disease and its mechanisms like cell trafficking, it is essential to implement the *in vitro* models technologies with organ-specific human-derived cells. Thus, to recapitulate the BM environment in an *in vitro* model it is essential to include: (i) a monolayer of ECs with a fluid flow applied on it to mimic the blood flow; (ii) the presence of perivascular cells surrounding the ECs such as pericytes, and the immune components (macrophages and/or neutrophils); (iii) all components need to be human and derived from the BM or that display BM-specific characteristics. Among the thirty-three literature works considered in this review, none include all these characteristics together. In the work of Jeon et al. (124) the shear stress was applied to the ECs layer and the model included almost all the cellular components necessary to replicate the vascular niche, however, the ECs were not BM-derived and the immune component originates from mice. As Jeon et al., all the 2D and 3D human BM vasculature *in vitro* models described, except for the works of Rafii et al. (106) and Aleman et al. (135), hold ECs derived from other vascular districts. Therefore, there is a need for a more standardized protocol for human bone marrow endothelial and perivascular cell isolation, or iPS-derived cell differentiated in BM cells to improve these advanced *in vitro* models of BM vasculature. Success in the combination of these advanced *in vitro* BM vasculature models with human/patients-derived cells will allow the study of physiological and pathological mechanisms that can be relevant to pre-clinical studies. Furthermore, these new generation of *in vitro* models can be used to investigate the effects of drugs or other treatments on cellular trafficking at the BM vasculature, paving the way for more effective treatments for cancer and cardiovascular diseases.
